# Antioxidant and Antiproliferative Activities of Heated Sterilized Pepsin Hydrolysate Derived from Half-Fin Anchovy (*Setipinna taty*)

**DOI:** 10.3390/md9061142

**Published:** 2011-06-23

**Authors:** Ru Song, Rongbian Wei, Bin Zhang, Zuisu Yang, Dongfeng Wang

**Affiliations:** 1 College of Food Science and Pharmacy, Zhejiang Ocean University, Zhoushan 316000, China; E-Mails: zhangbin_ouc@yahoo.com.cn (B.Z.); susan_70@yahoo.cn (Z.Y.); 2 College of Food Science and Engineering, Ocean University of China, Qingdao 266003, China; E-Mail: wangdf@ouc.edu.cn; 3 College of Marine Science, Zhejiang Ocean University, Zhoushan 316000, China; E-Mail: windswingcn@yahoo.com.cn

**Keywords:** half-fin anchovy pepsin hydrolysate, heated sterilization, antioxidant activity, antiproliferative activity, chemical profile

## Abstract

In this paper we studied the antioxidant and antiproliferative activities of the heated pepsin hydrolysate from a marine fish half-fin anchovy (HAHp-H). Furthermore, we compared the chemical profiles including the amino acid composition, the browning intensity, the IR and UV-visible spectra, and the molecular weight distribution between the half-fin anchovy pepsin hydrolysate (HAHp) and HAHp-H. Results showed that heat sterilization on HAHp improved the 1,1-diphenyl-2-picryl-hydrazil (DPPH) radical-scavenging activity and reducing power. In addition, the antiproliferative activities were all increased for HAHp-H on DU-145 human prostate cancer cell line, 1299 human lung cancer cell line and 109 human esophagus cancer cell line. The contents of free amino acid and reducing sugar of HAHp-H were decreased (*P* < 0.05). However, hydrophobic amino acid residues and the browning intensity of HAHp-H were increased. FT-IR spectroscopy indicated that amide I and amide III bands of HAHp-H were slightly modified, whereas band intensity of amide II was reduced dramatically. Thermal sterilization resulted in the increased fractions of HAHp-H with molecular weight of 3000–5000 Da and below 500 Da. The enhanced antioxidant and antiproliferative activities of HAHp-H might be attributed to the Maillard reaction.

## 1. Introduction

Heated sterilization is a common operation in food processing, and this process induces a variety of physical and biochemical changes that influence the nutritional and functional values of products, as well as the acceptable textures. During thermal processing of protein or hydrolysate-rich foods, the Maillard reaction occurs at high temperature [1,2]. In recent years, Maillard reaction products (MRPs) have been increasingly studied. MRPs have been reported to possess multiple-bioactivities, including antioxidant [3,4], antibacterial [5], antihypertensive [5], antimutagenic [6] and antiradical activities [7]. Moreover, MRPs have been recognized as an important flavoring enhancer for heat-processed food products [8].

Most MPRs were prepared through single pure amino acid, peptide or protein reacting with different reducing sugars [4,9,10]. Only a few studies have investigated the MPRs from peptides, hydrolysates, or proteins [1,2]. In our previous study we found that the pepsin hydrolysate of the marine fish half-fin anchovy (HAHp) was composed of small peptides, and demonstrated *in vitro* antioxidant and antiproliferative activities [11]. However, the activity changes for HAHp after thermal sterilization were not investigated. Thus, in this study we measured the antioxidant and antiproliferative activities of heated sterilized HAHp. Furthermore, the chemical profiles between HAHp and HAHp-H were compared, aiming at revealing the possible reasons for these enhanced bioactivities.

## 2. Results and Discussion

### 2.1. *In Vitro* Antioxidant Activity

**Figure 1:**
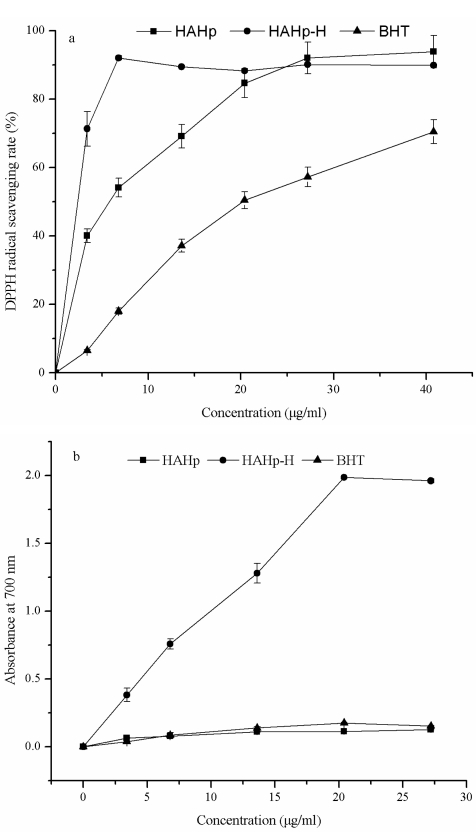
Comparison of antioxidant activities between half-fin anchovy pepsin hydrolysate (HAHp) and heated pepsin hydrolysate from a marine fish half-fin anchovy (HAHp-H). (**a**) DPPH radical-scavenging activity; (**b**) Reducing power. The concentration of HAHp and HAHp-H was represented as protein concentration. Each value was expressed as mean ± standard deviation (*n* = 3).

The antioxidant activities of HAHp, HAHp-H and butylated hydroxytoluene (BHT) were compared in Figure 1. HAHp-H exhibited a strong DPPH radical-scavenging activity at a concentration of 6.80 μg/mL and kept stable above that concentration (Figure 1a). By comparison, HAHp and BHT showed the increasing DPPH radical-scavenging activity in a concentration-dependent manner. The 50% scavenging concentrations (ED_50_ value) of HAHp, HAHp-H and BHT were 4.46 μg/mL, 1.19 μg/mL and 22.78 μg/mL, respectively. The lower the ED_50_ value, the higher the DPPH scavenging activity was. Obviously, HAHp-H had a stronger DPPH radical-scavenging activity than HAHp.

The reducing power of HAHp-H increased with increasing concentration (Figure 1b). At a concentration of 27.20 μg/mL, the absorbance at 700 nm reached 1.984, thereafter, the absorbance did not change significantly.

### 2.2. Antiproliferative Activity

It can be shown that both HAHp and HAHp-H were able to inhibit the proliferation of DU-145 human prostate cancer cell line, 1299 human lung cancer cell line and 109 human esophagus cancer cell line (Table 1). In addition, HAHp and HAHp-H displayed a dose-dependent manner on the antiproliferation of DU-145 and 1299 cell lines. The inhibitory rate of HAHp-H on the DU-145 cell line was significantly higher than that of HAHp (*P* < 0.05) at the same concentration, with an IC_50_ value of 13.67 mg/mL, about three-fold stronger than that of HAHp (IC_50_: 41.67 mg/mL). In contrast, there was no significant difference (*P* < 0.05) on the antiproliferation of 1299 human lung cancer cell line for HAHp and HAHp-H at concentrations ranging from 5 to 20 mg/mL. However, at a concentration of 40 mg/mL, the inhibitory rate of HAHp-H (95.68%) was significantly higher than that of HAHp (46.06%) (*P* < 0.05). The IC_50_ values of HAHp-H and HAHp on 1299 human lung cancer cells were 25.17 mg/mL and 40.28 mg/mL, respectively.

As for the antiproliferation of 109 human esophagus cancer cells, both HAHp and HAHp-H indicated inhibitory effects at a high concentration of 40 mg/mL. Moreover, HAHp-H showed stronger antiproliferative activity than HAHp (*P* < 0.05). Different types of cancer cells might have different cell membrane composition, fluidity and surface area [12], therefore, HAHp-H demonstrated variability in antiproliferative activities at the same concentration. The antiproliferative activity of fish hydrolysates or peptides on cancer cell lines was less studied compared with their other bioactivities. Picot *et al.* [13] reported fish protein hydrolysates from three blue whitings, three cods, three plaices and one salmon, showed significant antiproliferation on MCF-7/6 and MDA-MB-231 human breast cancer cell lines. Peptides were mainly responsible for these activities. Lee *et al.* [14] and Hsu *et al.* [15] isolated hydrophobic anticancer peptides from anchovy sauce and tuna dark muscle byproduct hydrolysates, respectively. To our knowledge, this is the first time that the antiproliferative activity of the pepsin hydrolysate of half-fin anchovy and its heated sterilization products (HAHp-H) have been reported.

### 2.3. Chemical Profiles

#### 2.3.1. Changes in Free Amino Acid and Reducing Sugar Levels

**Figure 2:**
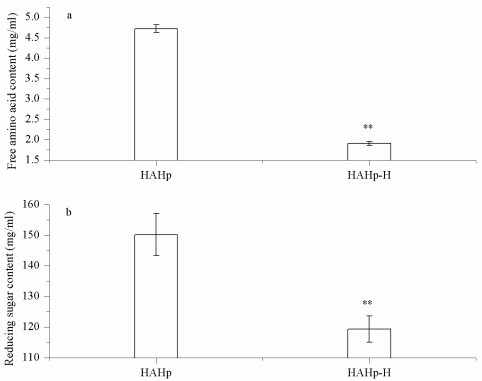
Comparison of free amino acid and reducing sugar levels between HAHp and HAHp-H. (**a**) Free amino acid content; (**b**) Reducing sugar content. Each value was expressed as mean ± standard deviation (*n* = 3).

As shown in Figure 2a, the content of free amino acid of HAHp was decreased from 4.72 to 1.91 mg/mL (*P* < 0.05) after heat sterilization (121 °C, 30 min). In addition, the reducing sugar content of HAHp-H was reduced to 119.3 mg/mL, statistically lower than that of HAHp (150.2 mg/mL) (Figure 2b). During thermal processing, peptides/free amino acids in hydrolysates interacted directly with reducing sugar (cross-linking) to form larger molecules, resulted in the reduced free amino acid and reducing sugar contents. On the other hand, higher molecular weight of peptides could degrade to smaller peptides or free amino acids [2,16]. The balance between peptides/amino acids cross-linking and peptides degradation determined the free amino acids content in the heated products. The result of Figure 2 indicated that reducing sugar or its degraded products attached to free amino acids (cross-linking) played an important role in HAHp-H.

#### 2.3.2. Amino Acid Analysis

It was observed that the sterilization product of HAHp had slightly higher total amino acid content compared with HAHp, this result was similar to the observations of Oluwaniyi *et al.* [17] that the processes of boiling (100 °C, 10 min) and roasting (145 °C, 15 min) increased the total amino acid contents of four marine fishes including herring, Atlantic mackerel, horse mackerel and white hake. As seen in Table 2, cysteine was lost completely in HAHp-H. Additionally, the content of lysine was decreased from 0.961 to 0.814 mg/100 mg. Since cysteine has the ability to form a dehydroalanyl residue and bind with the ɛ-amino group of lysine in the Maillard reaction [18], result of Table 2 implied that cysteine and losses of lysine under the heat treatment probably contribute to form cysteine-derived crosslink compounds, e.g., lysinoalanine. The levels of amino acid, *i.e.*, valine, leucine, phenylalanine, histidine and arginine, were all increased. In particular, the histidine level of HAHp-H was dramatically increased from 0.294 to 1.368 mg/100 mg. An interesting finding was that these amino acid levels, except for the losses of cysteine and lysine, were reported to be increased or damaged in different protein, peptide, or free amino acid/sugar heating systems [4,17]. We conjectured that differences in substrates and heating conditions determined amino acid composition in the thermal products.

Table 2, clearly shows that the contents of alanine, valine, leucine, arginine and histidine amino acids accounting for the presence of antioxidant activity [19], were slightly or dramatically increased in HAHp-H. A higher ratio of hydrophobic amino acids was also reported to improve the antioxidant activities of the Maillard reaction products (MPRs) [19,20]. Furthermore, the hydrophobic amino acid residues were important for the formation of hydrophobic tail in the COOH-terminal region, which exhibited a predominant role for anticancer peptides to mediate their cytotoxic effect [21]. The hydrophobic amino acids content of HAHp-H were increased to 8.947 mg/100 mg with a relative percent of 48.74%, indicating that HAHp-H should display higher antioxidant ability and antiproliferative activity than HAHp. Results shown in Figure 1 and Table 1 confirmed this viewpoint.

#### 2.3.3. UV-Visible Spectra and Browning Intensity

**Figure 3:**
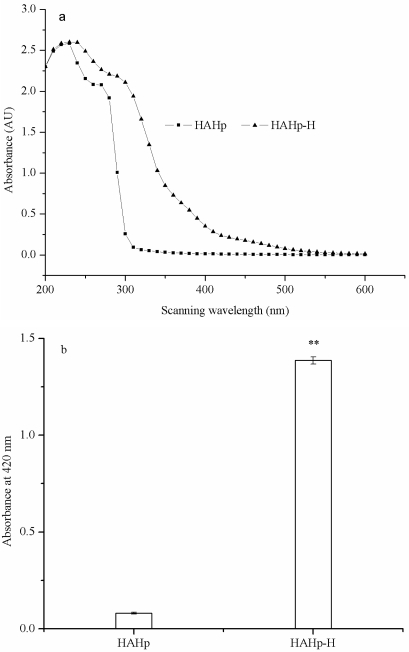
Comparison of UV-visible spectra and browning intensity between HAHp and HAHp-H. (**a**) UV-visible spectra; (**b**) Browning intensity, data in Figure 1b were expressed as mean ± standard deviation (*n* = 3).

The UV-visible spectra of HAHp and HAHp-H showed a similar contour. However, the absorption values of HAHp-H were generally higher than that of HAHp under same wavelength (Figure 3a). Sugar caramelization might occur in addition to the Maillard reaction when heated at temperatures above 120 °C or 9 < pH < 3, leading to a browning of the mixture [1,22]. However, provided amino compounds such as amino acids, peptides and proteins were present, the Maillard reaction usually took place [22]. In Figure 3a, HAHp-H had a maximum absorbance appeared in the range of 260–320 nm, which was characteristic of melanoidins [23,24]. The absorbance under 420 nm (browning intensity) was often used as an indicator for browning development in the Maillard reaction [25]. As can be seen in Figure 3b, the browning intensity of HAHp-H was increased to 1.386, significantly higher than that of HAHp (0.081) (*P* < 0.05), suggesting that the Maillard reaction might play a predominant role in HAHp-H. The results of Figure 3 were in accordance with the decreased free amino acid and reducing sugar contents in HAHp-H (seen in Figure 2).

#### 2.3.4. FT-IR Measurement

The vibration regions of the amide bonds of proteins and the chemical fingerprints of carbohydrates were readily identified in FT-IR spectroscopy [26,27]. The most distinctive spectral features for proteins were amide I band at 1600–1700 cm^−1^ (C═O stretching), amide II band at 1500–1550 cm^−1^ (N–H deformation) and amide III band at 1200–1300 cm^−1^ (C–N stretching and N–H deformation). Normally, the amide I band was strong, the amide II band was weak and the amide III band was moderate [26]. A series of overlapping peaks located in the region of 1180–953 cm^−1^, which were described as the “saccharide” bands resulting from vibration modes such as the stretchings of C–C and C–O and the bending mode of C–H bonds. These absorptions were weak in the spectra of most proteins [27].

**Figure 4:**
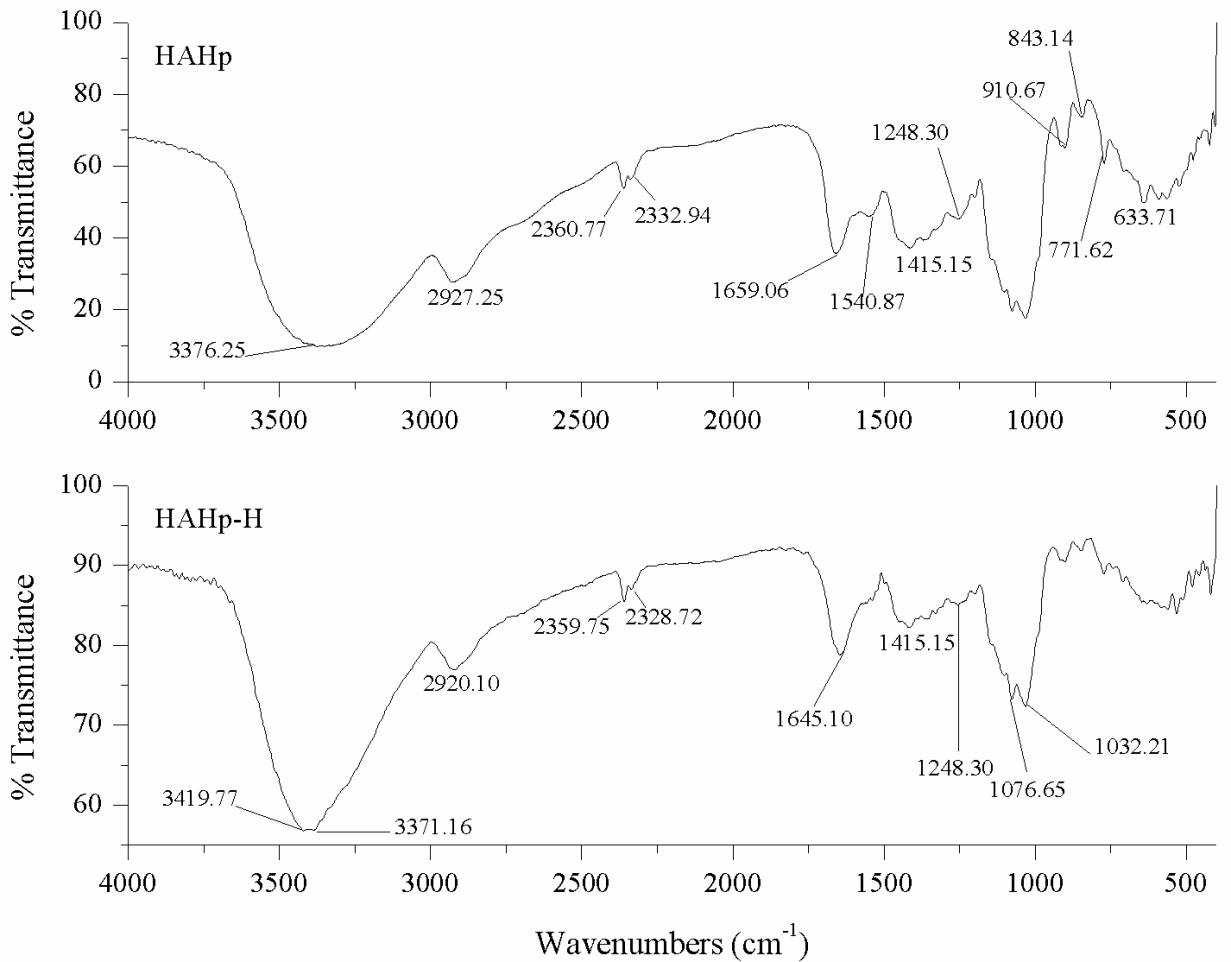
Comparison of FT-IR between HAHp and HAHp-H.

As can be seen in Figure 4, the bands of amide I in HAHp and HAHp-H were located at 1659.06 cm^−1^ and 1645.10 cm^−1^, respectively. The result meant that the amide I band of HAHp-H was modified by thermal sterilization, meanwhile, the result of Figure 4 also indicated that both HAHp and HAHp-H might contain the second structure of alpha-helical, which has a peak maximum around 1660–1650 cm^−1^ [28]. The IR spectral features of HAHp and HAHp-H between 1500 cm^−1^ and 1550 cm^−1^ showed a clear change and the band intensity of HAHp-H at 1540.87 cm^−1^ was decreased dramatically, indicating that heat sterilization resulted in a big conformation change of amide II band (N–H deformation). As for amide III bands, which were very complex in proteins, there was slight variation from the range of 1200 cm^−1^ to 1300 cm^−1^ region (Figure 4). Nevertheless, the FT-IR spectroscopy would not be sufficiently sensitive to distinguish such small changes.

In the IR region of 1180–953 cm^−1^, which associated with “saccharide” bands, the absorbances of HAHp and HAHp-H were not weak, implying that saccharides were contained in HAHp and HAHp-H. The result of FT-IR could contribute to explain the reason for MRPs formation in HAHp-H even without addition of sugars. In the mid-infrared spectrum of MRPs, several chemical structures were changed. For example, the group of NH_2_, especially from lysine, was lost. While the amount of groups, including the Amadori compound (C═O), Schiff base (C═N) and pyrazines (C═N) associated with Maillard products might be increased [4].

#### 2.3.5. Molecular Weight (MW) Distribution

The MW profiles of HAHp and HAHp-H were displayed in Table 3. HAHp was a mixture of small peptides and the percent of peptides below 3000 Da was above 95%, while in HAHp-H it decreased to 60.77%. The decreased fractions were within the ranges of 1000–3000 Da and 500–1000 Da. However, the higher MW of 3000–5000 Da and lower MW below 500 Da in HAHp-H were increased to 38.06% and 19.00%, respectively (*P* < 0.05). The result was similar to the study of Liu *et al.* [3]. Because of the presence of peptides/amino acids cross-linking in the Maillard reaction, larger molecules tended to form, while peptides/proteins degradation resulted in the increased smaller peptides or free amino acids. High MW and low MW components in MRPs were accountable for the enhanced antioxidant activities [29], and hydroxyl groups of MRPs were effective electron donors to increase reducing power [30].

## 3. Experimental Section

### 3.1. Preparation of Heated Products from Half-Fin Anchovy Pepsin Hydrolysate

Pepsin hydrolysate of half-fin anchovy (HAHp) was prepared as the optimized method described previously [31]. The soluble peptides content and degree of hydrolysis of HAHp were (81.78 ± 0.04)% and (18.12 ± 0.39)%, respectively, determined by the following methods [32]. Briefly, HAHp was added to 15% of trichloroacetic acid (TCA) at a ratio of 1:1. The mixture was maintained for 30 min and centrifuged (8000 rpm/min, 15 min). The total soluble peptides content was computed as the proportion (%) of total nitrogen content in the supernatant with respect to the total nitrogen content in HAHp. The degree of hydrolysis was evaluated as the proportion (%) of total free amino nitrogen in the supernatant with respect to the total nitrogen content in fish muscle. The contents of total nitrogen and free amino nitrogen were assayed through micro Kjeldahl and ninhydrin reaction methods, respectively.

HAHp was heated for 30 min at 121 °C to imitate food steaming sterilization procedure. The thermal products of HAHp were designated as HAHp-H. Some portions of HAHp-H were also freeze-dried to obtain powder for further amino acid composition analysis and FT-IR spectroscopy. Protein concentrations of HAHp and HAHp-H were determined using Bradford’s method [33].

### 3.2. *In Vitro* Antioxidant Activity

#### 3.2.1. DPPH Radical-Scavenging Assay

The free radical scavenging activity was determined by the 1,1-diphenyl-2-picryl-hydrazil (DPPH) method with few modifications [34]. Briefly, 375 μL of 0.02% DPPH solution in 99.5% ethanol was added to 1.5 mL of HAHp and 1.5 mL of 99.5% ethanol, then mixed thoroughly and kept in the dark at room temperature for 60 min. The absorbance of the mixtures was measured at 517 nm. HAHp and butylated hydroxytoluene (BHT) were tested in the same way for comparison. DPPH radical scavenging activity was calculated as follows:

Radical scavenging activity (%)=[(control−sample+blank)/control]×100

where control, replaced 1.5 mL of sample with 1.5 mL of distilled; blank, replaced 375 μL of 0.02% DPPH with 375 μL of 99.5% ethanol.

#### 3.2.2. Reducing Power Assay

The presence of reductants of samples could result in reducing Fe^3+^/ferricyanide complex to the ferrous form (Fe^2+^), and the Fe^2+^ could therefore be monitored by measuring the formation of Perl’s Prussian blue at 700 nm. A higher absorbance at 700 nm meant a better reducing power [30]. The reducing power of HAHp-H was measured according to the method of Oyaizu [35] with slight modifications. 1.0 mL of HAHp-H was mixed with 1.0 mL of 0.2 M sodium phosphate buffer (pH 6.6) and 1.0 mL of 1% potassium ferricyanide (K_3_Fe(CN_6_)). The mixture was incubated at 50 °C for 20 min, followed by addition of 1.0 mL of 10% trichloroacetic acid. Then, the mixture was centrifuged at 3000 g for 10 min and 2.0 mL of supernatant was blended with 2.0 mL of deionized water and 0.8 mL of 0.1% ferric chloride (FeCl_3_). The absorbance of the mixture was measured at 700 nm. The reducing power of HAHp and BHT were also determined for comparison.

### 3.3. Antiproliferative Activity

#### 3.3.1. Cell Lines and Cell Culture

Three human cancer cell lines, DU-145 prostate cancer cell line, 1299 lung cancer cell line and 109 esophagus cancer cell, were provided by College of Food Science and Pharmacy, Zhejiang Ocean University and cultured in RPMI 1640 medium at 37 °C in a humidified atmosphere with 5% CO_2_ incubator (ThermoForma, USA), supplemented with 10% heat-inactivated fetal calf serum, 200 mM l-glutamine, 100 units/mL of penicillin, and 100 units/mL streptomycin.

#### 3.3.2. MTT Assay

The inhibition rate of cancer cell lines after treatment with HAHp and HAHp-H was determined by the MTT method [36]. Cancer cell lines were transferred to fresh media every 2–3 days to maintain in an exponential growth and digested by 0.25% trypsin to obtain cell suspension. Then, 200 μL of cell suspension (1 × 10^4^ cells/mL) was transferred into a 96 wells plate and incubated for 24 h. After this period, the suspension was removed and 200 μL of the sample (HAHp and HAHp-H, concentration ranging from 5 mg/mL to 40 mg/mL) was added, followed by incubation for 48 h at 37 °C. Then, 200 μL of MTT (1 mg/mL) was added to these cells respectively and incubated for 4 h at 37 °C. The medium was removed and 150 μL of DMSO was added to dissolve the formazan crystals formed and precipitated. Finally, the absorbance was read at 490 nm with an ELISA reader (BR680, USA). The inhibition rate was calculated using the following equation:

$$\text{Inhibition}\;\text{rate}\;(\%)=[({\text{A}}_{\text{C}}-{\text{A}}_{\text{S}})/({\text{A}}_{\text{C}}-{\text{A}}_{\text{B}})]\times 100$$

where A_C_, absorbance of control, using 200 μL of RPMI 1640 complete medium instead of 200 μL of HAHp-H; A_S_, absorbance of sample; A_B_, absorbance of RPMI 1640 complete medium.

### 3.4. Measurement of Chemical Profiles

#### 3.4.1. Free Amino Acid Content

The content of free amino acids was determined by the method of ninhydrin reaction [37]. 0.2 mL of sample was mixed with 3.8 mL of distilled water, 1.0 mL of ninhydrin and 1.0 mL of 0.2125 mol/L sodium phosphate buffer (pH 8.2) in a 25 mL of cylinder. The solution was heated at 100 °C for 15 min and cooled down to room temperature quickly. Then, the volume of the mixture was adjusted to 25 mL by addition of distilled water. After being kept at room temperature for 15 min, the absorbance of the mixture was measured at 570 nm. A calibration curve was obtained by using l-alanine as a standard. The content of free amino acids was expressed as mg per mL of sample.

#### 3.4.2. Reducing Sugar Level

Reducing sugar levels of HAHp and HAHp-H were determined by the Fehling method with some modifications [38]. Briefly, HAHp and HAHp were diluted to 100 mL after removal of proteins by addition of zinc acetate and potassium ferrocyanide solution. 5 mL of Fehling solution A (15 g copper sulfate and 0.05 g methylene blue dissolved in distilled water), 5 mL of Fehling solution B (50 g sodium potassium tartrate tetrahydrate, 75 g sodium hydroxide and 4 g potassium ferrocyanide dissolved in distilled water) and 10 mL of distilled water were added into a 250 mL of conical flask. The mixture was heated to boilling in 2 min, followed by titration with HAHp and HAHp to reach bright yellow colour. The consumed volumes of HAHp and HAHp-H were recorded, respectively. HAHp and HAHp-H were replaced by glucose, which was used as the standard of reducing sugar, to standardize the mixture of Fehling solution A and B under the same condition. The content of reducing sugar was expressed as mg per mL of sample.

#### 3.4.3. Amino Acid Analysis

HAHp and HAHp-H powders were respectively dissloved in 6 mol/L HCl and hydrolyzed at 110 °C for 24 h. After removal of HCl under vacuum, the dried samples were then redissolved in 0.1 M sodium citrate buffer (pH 2.2). Subsequent amino acid analysis was carried out in an automatic amino acid analyzer (Hitachi 835-50, Tokyo, Japan). Results were determined as mg per 100 mg of sample.

#### 3.4.4. UV-Visible Spectra and Browning Intensity

UV-visible spectra of HAHp and HAHp-H were recorded by a Shimadzu 1200UV-vis spectrophotometer (Tokyo, Japan) with the wavelength ranging from 200 to 600 nm. The browning intensity was measured at 420 nm [39].

#### 3.4.5. Fourier Transform Infrared Spectroscopy (FT-IR) Measurement

All IR spectra of HAHp and HAHp-H were detected using an FT-IR spectrometer (NICOLET NEXUS670, DTGS). HAHp and HAHp-H powders were mixed with potassium bromide (KBr) powder separately. Scanning was carried out in the range of 4000–400 cm^−1^ with a resolution of 4 cm^−1^ and 32 scans were calculated to obtain an average for each sample.

#### 3.4.6. Molecular Weight (MW) Distribution

The MW distribution profiles of HAHp and HAHp-H were estimated by gel permeation chromatography (GPC) using an ÄKTA purifier system, equipped with a Superdex-75 HR 10/300 column from Amersham Pharmacia, Uppsala, Sweden. The elution consisting of 50 mM sodium phosphate buffer (pH 5.8 containing 0.15 mol/L NaCl) was delivered at a flow rate of 0.4 mL/min. 100 μL of sample (filtered by a 0.22 μm mircofiltre) was injected onto the column. Chromatograms were recorded by UV detection at 280 nm. Four MW standards, including bovine serum albumin (68,000 Da), aprotinin (6512 Da), vitamin B_12_ (1355 Da) and oxidized glutathinone (613 Da), were used to obtain a MW calibration curve. The data analysis was performed using gel permeation chromatography software.

#### 3.4.7. Statistical Analysis

Data were represented as mean ± standard deviation. The significant differences (*P* < 0.05) between samples were evaluated through the student’s *t*-test.

## 4. Conclusions

In this study, the process of sterilization on HAHp had desirable effect on the antioxidant and antiproliferative activities. The results of chemical profiles revealed that it is likely that the Maillard reaction occurred in HAHp-H. Moreover, the higher content of hydrophobic amino acids and the formation of specific higher and lower molecular weight components in HAHp-H might be accountable for the enhanced antioxidant and antiproliferative activities. To summarize, heated sterilization treatment should be encouraged as a convenient approach for HAHp to improve the bioactivity in consumption.

## Figures and Tables

**Table 1:** Comparison of antiproliferative activity between HAHp and HAHp-H ^a^.

**Cell lines**	**Samples**	**Concentration (mg/mL)**			
		**5**	**10**	**20**	**40**
DU-145 human prostate cancer cell	HAHp	0.21 ± 0.11 ^aA^	8.39 ± 0.11 ^bA^	23.36 ± 0.69 ^cA^	44.40 ± 0.74 ^dB^
	HAHp-H	17.31 ± 0.28 ^aC^	50.07 ± 0.76 ^bB^	71.28 ± 6.21 ^cB^	98.81 ± 0.34 ^dC^
1299 human lung cancer cell	HAHp	–	4.47 ± 2.05 ^aA^	20.62 ± 5.70 ^bA^	46.06 ± 0.95 ^cB^
	HAHp-H	4.92 ± 1.10 ^aB^	9.27 ± 2.21 ^aA^	21.31 ± 7.93 ^bA^	95.68 ± 3.68 ^cC^
109 human esophagus cancer cell	HAHp	–	–	–	29.90 ± 7.18 ^A^
	HAHp-H	–	–	–	55.99 ± 6.26 ^B^

a Data were shown as mean ± standard deviation of five replicates. Different letter superscripts (a–d) represented significant difference in the same row (*P* < 0.05). Different capital letter superscripts (A–C) represented significant difference in the same column (*P* < 0.05).

**Table 2:** Comparison of amino acid composition between HAHp and HAHp-H.

**Amino acid**	**HAHp (mg/100 mg)**	**Relative percent (%)**	**HAHp-H (mg/100 mg)**	**Relative percent (%)**
Aspartic acid	1.808	10.32	0.000	0.00
Threonine	0.755	4.31	1.413	7.70
Serine	0.859	4.90	0.780	4.25
Glutamic acid	2.393	13.66	2.256	12.29
Glycine	1.298	7.41	1.288	7.02
Alanine	2.327	13.28	2.535	13.81
Cysteine	0.157	0.90	0.000	0.00
Valine	1.480	8.45	1.812	9.87
Methionine	0.783	4.47	0.819	4.46
Isoleucine	0.584	3.33	0.818	4.46
Leucine	0.922	5.26	1.192	6.49
Tyrosine	0.170	0.97	0.589	3.21
Phenylalanine	0.303	1.73	0.326	1.78
Lysine	0.961	5.49	0.814	4.43
Histidine	0.294	1.68	1.368	7.45
Arginine	0.778	4.44	0.903	4.92
Proline	1.645	9.39	1.445	7.87
Tryptophan	ND ^a^	ND ^a^	ND ^a^	ND ^a^
ΣAA ^b^	17.518	100	18.357	100
ΣHAA ^c^	8.044	45.92	8.947	48.74

a ND, not determined;

b ΣAA represented the total content of amino acids;

c ΣHAA represented the total content of hydrophobic amino acids.

**Table 3:** Comparison of molecular weight distribution between HAHp and HAHp-H ^a^.

**MW (Da)**	**>5000**	**3000–5000**	**3000–1000**	**1000–500**	**<500**
HAHp	4.29 ± 0.35 ^a^	0.50 ± 0.16 ^a^	53.57 ± 1.82 ^a^	31.67 ± 0.25 ^a^	9.99 ± 1.22 ^a^
HAHp-H	1.63 ± 0.67 ^b^	38.06 ± 0.15 ^b^	24.74 ± 1.49 ^b^	17.03 ± 0.11 ^b^	19.00 ± 0.06 ^b^

a Data in the table were mean of relative areas ± standard deviation of duplicate analyses. Different superscripts in the same column indicated significant difference (*P* < 005).
